# Supplementary material for: Patterns of Intron Gain and Loss in Fungi
Source: PLoS Biol. 2004 Nov 30;2(12):e422. doi: 10.1371/journal.pbio.0020422 (PMC532390; doi:10.1371/journal.pbio.0020422)
Supplement: Table S1 — Also available at http://genes.mit.edu/NielsenEtAl/. (4.3 MB ZIP). [file pbio.0020422.st001.zip › NielsenEtAl/html/554.html]

AN3757.1.NCU03276.1.MG00142.1.FG07077.1


```
 CLUSTAL W (1.82) Multiple Sequence Alignments - Introns Inserted


Sequence 1: NCU03276.1	320 aa
Sequence 2: MG00142.1	263 aa
Sequence 3: FG07077.1	402 aa
Sequence 4: AN3757.1	286 aa
Alignment Length: 422 aa
Number Identitical Residues: 123 aa
Alignment Score (without introns) 6707


MG00142.1 	---------------------------------------~--------------------
NCU03276.1	MSSPSVTPPAPDGNSFLVQIVSLLSSAASHLRLPVIAST0GVAAILTGLLYFKQ------
FG07077.1 	-MSSSNSPASQSSNNYVEQTVSVLSSVASYMRLPALAST~VRTPKLLALKSQSQNQRPRP
AN3757.1  	---------------------------------------~--------------------
          	                                                            

MG00142.1 	------------------------------------------------------------
NCU03276.1	------------------------------------------------------------
FG07077.1 	QSSPDTDINALLHRVSLPSSPRYYTLSKSPYTTPLVSISISISVPVFFLSLPPFHYCSTR
AN3757.1  	------------------------------------------------------------
          	                                                            

MG00142.1 	--------------~-------MPPNSRTQVSRPSQFNIKDFEELMIPTPDGEKLSAFYI
NCU03276.1	-------------K2ALIYPSHMPPGSRTDIPRPSFYRIKDYEELIIPTKDGEKLSAFYI
FG07077.1 	IIILPRPALTTCIR~ALIYPSNIPANSRSDVPKPSDFGLSNFEELYIPTDDGEKLSAFYI
AN3757.1  	-----------MCS~ELIYPRNIPADARTNVPKPRQFGIEDYEELQLRTPDGESLHAYFI
          	                   .  :*..:*:::.:*  : :.::*** : * ***.* *::*

MG00142.1 	RGSRNGRNSNVTIIMFHGNAGNIGHRLPIARHLVELMGCNVFMLEYRGYGLSTGTADESG
NCU03276.1	RGPKGGSNSKVTVIMFHGNAGNIGHRLPIARMLLQAAGCNIFMLEYRGYGISTGHPDESG
FG07077.1 	RGPRGHKNSNITILMFHGNAGNIGHRLPIARMIINYIGCNVFMLEYRGYGSSTGQPDESG
AN3757.1  	RAPRKRVDQNLTVLMFHGNAGNVGHRIPIAKIMQDYLGCHVLMLEYRGYGLSTGVPDEHG
          	*..:   :.::*::********:***:***: : :  **:::******** *** .** *

MG00142.1 	LMIDAQTGLDYLRDRPETRKHRLVVYGQSLGGSVAIRLVSKNQAAGDIVGLILENTFLSM
NCU03276.1	LNIDAQTALDYLRDRAETRDHKYIVYGQSLGGAVSVKLVSKNQGRGDIAGLILENTFLSM
FG07077.1 	LNIDAQTGLNYLRQRAETRDHKLMVYGQSLGGAVAIKLVSKNQEAGDIAGLILENTFLSI
AN3757.1  	LKIDAQTALDHLRLRGETANSRIVVYGQSLGGAVAINLVANNEDKGSISGLILENTFLSI
          	* *****.*::** * ** . : :********:*::.**::*:  *.* **********:

MG00142.1 	RKLIP-------------------SVIPPTKYFAFLCHQVWPSDVAIPNITKVPILFLSG
NCU03276.1	RKLIP-------------------SIIPPARYLASLCHQVWATDTLIGNV-KVPTLFLSG
FG07077.1 	RKLIP-------------------SVVPPAKYLTLLCHQVWPSESVLPNITKVPTLFISG
AN3757.1  	RKLIPRHVPSSISASNICTMITISSVFPPARYLARFCHQTWTSEEVLPKITKTPILFLSG
          	*****   .:: :::. .:  : :*:.**::*:: :***.*.::  : :::*.* **:**

MG00142.1 	QQDEIVP2PSHMRQLYELSAAPNKIWKPLPNGDHNSSVLEDGYFDAISDFVASVASEPPR
NCU03276.1	LQDEIVP2PTHMKQLYHLSNAPIKIWKPLPNGDHNSSVLEEGYFEAIAEFIQRVSSGDP-
FG07077.1 	LQDEIVP2PKHMKQLYEISAAPTKRWKPLPAGDHNSSVLEEGYFEAMSDFIAEVTDDQEK
AN3757.1  	LQDEIVP2PSNMTQLFAICNSKRKVWRTLPNGAHNDSVAEPDYFEHIHSFVTEEVLKHN-
          	 ****** *.:* **: :. :  * *:.** * **.** * .**: : .*:         

MG00142.1 	LSEKI
NCU03276.1	--EKK
FG07077.1 	EKTRS
AN3757.1  	-----
          	
```
